# LAYERING THE IMPACTS OF STRUCTURAL RACISM ON HEALTHY AGING TRAJECTORIES IN A LONGITUDINAL COHORT

**DOI:** 10.1093/geroni/igae098.2122

**Published:** 2024-12-31

**Authors:** Richard Sadler

**Affiliations:** Michigan State University, Flint, Michigan, United States

## Abstract

African Americans are more at risk for earlier and more pronounced cognitive and functional decline. Direct impacts of racism are responsible for disparities in medical quality, access, and treatment are documented drivers of these racial disparities in aging health inequities. But largely understudied is the role of the built and social environment as a foundational determinant of health. In particular, a web of elements of structural racism in the built environment (STRAßE) work together to create environments where amenities are scarce and disamenities proliferate. While many studies are keen to include redlining as one such measure, emerging evidence suggests that additional STRAßE variables (i.e. blockbusting, urban renewal, freeway construction) may be key in illuminating health linkages. Here we build such measures for the city of Baltimore for use in an expanded dataset of the Healthy Aging in Neighborhoods of Diversity Across the Life Span (HANDLS) epidemiological cohort study. Our team has proposed work with HANDLS to collect complete residential histories for ~800 midlife to older Baltimore-born men and women (ages 30-64 at baseline), tracked up to 13 years across three waves. We will illustrate spatial-temporal specific exposures of STRAßE and establish a framework for future work. We use GIS to digitize and geocode the extent of STRAßE and identify spatial-temporal overlaps. Initial findings show interesting links between key STRAßE measures (i.e. redlining lacking overlap with blockbusting, gentrification co-occurring with redlining and urban renewal), illustrating the importance of considering a wider range of measures than has been examined to date.

